# Influence of Concurrent Exercise Training on Ankle Muscle Activation during Static and Proactive Postural Control on Older Adults with Sarcopenic Obesity: A Multicenter, Randomized, and Controlled Trial

**DOI:** 10.3390/ejihpe13120192

**Published:** 2023-11-27

**Authors:** Elmoetez Magtouf, Sabri Gaied Chortane, Oussema Gaied Chortane, Sébastien Boyas, Bruno Beaune, Sylvain Durand, Wael Maktouf

**Affiliations:** 1 Research Laboratory (LR23JS01) «Sport Performance, Health & Society», Higher Institute of Sport and Physical Education of Ksar Saîd, University of “La Manouba”, Tunis 2010, Tunisia; moetaz.magtouf@outlook.com (E.M.); sabrigaied1@gmail.com (S.G.C.); oussama.gaeid@gmail.com (O.G.C.); 2 Laboratory “Movement, Interactions, Performance” (UR 4334), Department of Sport Sciences, Faculty of Sciences and Technologies, Le Mans University, 72000 Le Mans, France; sebastien.boyas@univ-lemans.fr (S.B.); bruno.beaune@univ-lemans.fr (B.B.); sylvain.durand@univ-lemans.fr (S.D.); 3 Bioengineering, Tissues and Neuroplasticity, UR 7377, Faculty of Health, University of Paris-Est Créteil, 8 rue du Général Sarrail, 94010 Créteil, France

**Keywords:** physical exercises, obesity, sarcopenia, static balance, proactive balance, ankle muscle activation

## Abstract

Sarcopenic obesity (SO), characterized by age-related muscle loss and excess body fat, significantly impairs postural control. However, limited research has explored the effects of concurrent exercise training on neuromuscular strategies during postural control in older adults with SO. The study enrolled 50 older adults with SO, split into an intervention group (IG, *n* = 25, mean age = 76.1 ± 3.5 years; mean BMI = 34.4 ± 4.0 kg/m^2^) and a control group (CG, *n* = 25, mean age = 75.9 ± 5.4 years; mean BMI = 32.9 ± 2.3 kg/m^2^). Participants in the IG were engaged in 60-min Total Mobility Plus Program (TMP) sessions three times a week for four months, while the CG maintained their typical daily activities. Standardized evaluations were conducted both before and after the intervention. These assessments included the Romberg and Timed Up and Go (TUG) tests, as well as the measurement of Center of Pressure (CoP) displacements parameters under various conditions. Additionally, ankle muscle activities were quantified during postural control evaluations and maximal voluntary contractions of plantar and dorsal flexors. Post-intervention results revealed a significant reduction of the standing time measured in the Romberg (−15.6%, *p* < 0.005) and TUG (−34.6%, *p* < 0.05) tests. Additionally, CoP area and velocity were notably reduced in various conditions (*p* < 0.05). Postural control improvements were associated with an increase of strength (*p* < 0.05) and decrease of ankle muscle activation (*p* < 0.05). These findings highlight the reversibility of neuromuscular system alterations associated with the synergistic effects of sarcopenia and obesity, emphasizing the trainability of postural control regulation within this population. By incorporating these insights into clinical practice and public health strategies, it seems possible to optimize the health and well-being of older adults with SO.

## 1. Introduction

Postural control is a cornerstone of daily life activities, forming the fundamental basis for a wide range of tasks [1]. The ability to maintain postural stability is not only essential for preventing falls but also for facilitating efficient and coordinated movements [2]. This complex process relies on several mechanisms, with a significant emphasis on the strength of the ankle mobilizing muscles and the coordination of neuromuscular activation [3]. The muscles surrounding the ankle joint function as stabilizers, furnishing the requisite force to uphold balance and counter external disturbances effectively [4]. Concurrently, the neuromuscular system precisely governs the activation and synchronization of these muscles, adapting seamlessly to alterations of the ground characteristics or the demands of various movements [5].

Alterations associated with sarcopenia can have a profound impact on the functioning of the neuromuscular system [6], potentially resulting in adverse effects on postural control [7,8]. This decline in neuromuscular function plays a pivotal role in predisposing older individuals to a heightened risk of falls [9,10]. Extensive research has thoroughly explored the influence of aging on postural control, consistently revealing a prevalent trend of increased center of pressure (CoP) displacement as individuals advance in age [4,11]. To counterbalance the diminishing efficiency of the musculotendinous system that naturally occurs with aging, there is a corresponding surge in the activity of postural muscles, particularly those encompassing the ankle joint during upright stance [12]. This heightened muscle engagement aims to bolster overall ankle stiffness through co-contraction, primarily involving the plantar and dorsal flexor muscles [4,13,14]. While this strategy proves beneficial in responding to transient disturbances, it also comes at the cost of escalated energy expenditure and premature fatigue, potentially jeopardizing postural stability and elevating the risk of falls [14,15].

It has been well established that obesity is associated with postural control alterations [2,16,17] due to the increased body mass of each body segment [18]. Postural control alterations in obese individuals could be explained by the anterior position of the center of pressure (CoP) from the axis of rotation (i.e., ankle joint) related to the accumulation of fat body mass in the abdominal area [18,19,20]. Consequently, obese individuals have to generate higher force at the ankle joint to counteract the forward position of CoP in order to avoid falls [20]. Other explanations of the altered postural control in obese individuals are related to altered proprioception process and increased ankle muscle activities. In fact, obesity is associated with low sensitivity of the plantar mechanoreceptors due to the high-pressure required to support the body mass [21]. In this context, Wa and Madigan [22] have reported that altered postural control parameters in obese individuals are related to a decreased sensitivity of the plantar mechanoreceptors. Altered postural control may also be related to increased ankle muscle activities [2]. Thus, higher muscle activities could induce earlier fatigue development in muscle extremities [23] because obesity is associated with earlier fatigue and delayed muscle recovery [24,25].

The combination of obesity and aging, often referred as sarcopenic obesity (SO), could dramatically reduce postural control abilities and capacities to perform daily living activities [4,6,8]. SO is characterized by a simultaneous presence of excess body fat and age-related muscle loss [26], and it has been shown to have a profound impact on various aspects of postural control, such as alterations in parameters like the speed and area of the CoP during static postural control testing [8,27]. In this context, Maktouf et al. [2] demonstrated that altered postural control in obese older adults may be related to an increase in plantar flexor activities and decreased force production capacities relative to the body mass.

To the best of our knowledge, no prior studies have examined the effects of a comprehensive physical activity program on neuromuscular strategies related to ankle joint function during both static and proactive postural control, particularly in the context of older adults with SO. The implementation of the Total Mobility Plus Program (TMP), designed to encompass a diverse spectrum of exercises targeting strength, balance, motor skills and mobility, is poised to comprehensively address these intricacies. One notable study by Maktouf et al. [28] focused on the impact of an adapted physical activity intervention on postural control parameters in obese older adults. However, their research has primarily concentrated on balance-related outcomes and lacks a comprehensive evaluation of the crucial neuromuscular elements that are essential for improving balance, such as muscle activation. Recognizing these gaps in the existing literature, it becomes increasingly apparent that a comprehensive investigation encompassing postural control regulation, coupled with a detailed exploration of the potential underlying neuromuscular factors driving improvements, is imperative.

This study seeks to assess the efficacy of the TMP program on postural control parameters and on the neuromuscular capacity of ankle muscles in older adults with SO. We hypothesize that the TMP program significantly improves postural control parameters and enhances the neuromuscular capacity of ankle muscles in this population. Furthermore, this investigation aims to explore the intricate relationship between the enhancements in postural parameters and the concurrent modifications in neuromuscular strategies. We hypothesize that enhancements in steady-state and proactive postural control parameters are positively correlated with a decrease in ankle muscle activities.

## 2. Materials and Methods

### 2.1. Experimental Design

This study was conducted following the Consolidated Standards of Reporting Trials (CONSORT) guidelines [29] and employed a single-blinded, prospective, controlled, randomized multi-center design, where participants were randomly assigned to either an intervention group (IG) or a control group (CG). The CG underwent pre- and post-evaluation tests without any intervention, while the IG engaged in a rigorous four month program with tri-weekly sessions. The recruitment phase spanned three weeks, with an additional one week screening phase, then three weeks allocated for experimental testing before the intervention, and another three weeks for experimental testing after the intervention. These assessments encompassed anthropometric measurements, clinical health evaluations, balance tests with electromyography assessment of ankle muscle activation, and maximal voluntary contraction tests of plantar and dorsal flexor muscles.

### 2.2. Recruitment and Randomization

Participants for this study were recruited from four distinguished Tunisian centers, each specializing in diverse aspects of obesity, between 1 March and 31 October 2022, through a combination of direct clinic recruitment and leaflet distribution. In order to qualify for participation, individuals were required to satisfy specific conditions, which encompassed having a body mass index (BMI) exceeding 30 kg/m^2^, a handgrip force (HF) lower than 17 N, a gait speed less than 1.0 m/s, and an age exceeding 65 years. Exclusion criteria encompassed the presence of neurological or cognitive impairments, severe cardiovascular issues, substantial musculoskeletal deformities or injuries in the lower extremities, concurrent medical conditions or chronic illnesses, and a Montreal Cognitive Assessment (MoCA) test score below 26. These eligibility requirements were ascertained through an extensive survey questionnaire and were subject to examination by the medical personnel.

Randomization between groups occurred on the day of inclusion and was overseen by the chief investigator at each center. Each participant was randomly assigned to one of the two groups: the CG or IG. The specifics of the TMP program were intentionally not disclosed to the participants to maintain blinding throughout the study. Instead, a blinded assessor conducted visits twice before and after the program, while an unblinded kinesiologist, who was knowledgeable about the TMP program, conducted separate visits during the program to provide treatment and exercise sessions. The randomization list was generated by an independent statistician from the Clinical Research Unit of our laboratory, who had no direct involvement in the study. For randomization, a computer-generated list was created using the Clinical Trial Randomization Tool (National Cancer Institute, Bethesda, MD, USA). This list was subsequently uploaded into an online case report form. Each study participant received a unique allocation study number in a sequential format (TMP00X), ensuring that the randomization process was both transparent and unbiased. Blinding was rigorously maintained until the database was finalized. To further enhance data integrity and accuracy, all necessary data stipulated by the study protocol were diligently entered into the Electronic Data Capture system in real time as they were acquired, ensuring contemporaneous record-keeping. Additionally, we employed the services of a clinical research associate with expertise in data management and adhered to principles of good clinical practice.

### 2.3. Intervention Program

The TMP program is reported using the Template for Intervention Description and Replication (TIDieR) guidelines and is based on the methodology presented in the study of Ferhi et al. [30] as demonstrated in Table 1.

### 2.4. Outcomes Measures

#### 2.4.1. Steady-State and Proactive Postural Control

The evaluation of static steady-state postural control was performed utilizing the Romberg (ROM) test. Participants were asked to maintain an upright position for 30 s without wearing shoes. They were instructed to keep their feet close together and extend their arms fully in front of their bodies, with palms facing upwards, while keeping their eyes closed. If participants opened their eyes, made arm or foot movements to regain stability, or needed assistance from the operator, the test was terminated. Each participant completed three trials, with a one-minute rest period between each trial, and the best-recorded standing time in seconds was noted.

To assess proactive postural control, we used the Timed Up and Go (TUG) test. Participants were directed to start by sitting in a chair with their arms resting on the armrests. They were then instructed to stand up from the chair, walk 3 m at their normal walking pace, turn around, and then sit back down. Two test trials were carried out, and the best time achieved in seconds was documented as the outcome measure.

#### 2.4.2. Anthropometric Measurement

Participants’ height (H) was accurately measured using a digital floor scale. Body mass (BM) and fat body mass (%) were assessed using an impedance meter (Tanita; SC 24, Amsterdam, The Netherlands). Body mass index (BMI, kg/m^2^), fat body mass (FBM, kg) and lean body mass (LBM, kg) were determined through the following formulas:

BMI (kg/m^2^) = BM (kg)/H^2^ (m^2^) (1)

FBM (kg) = body fat (%) × BM; and LBM (kg) = BM − FBM(2)

#### 2.4.3. Maximal Voluntary Contraction Testing Measurement

Maximal voluntary contractions (MVC) were measured utilizing a dynamometer (Sauter FL1K, Balingen, Germany) during isometric contractions of the ankle plantar flexor (PF) and dorsal flexor (DF) muscles [2]. Three trials were conducted for each condition with a 1 min rest between trials. For both MVC of PF and DF, the average of the highest value from the three trials was recorded. Relative force (MVC/BM) was then computed [30].

#### 2.4.4. Postural Control Evaluation

Postural control during quiet standing was assessed using a force platform (Zebris; FDM, Isny, Germany). Participants were directed to stand barefoot on the platform with their feet together and arms placed alongside their body. They performed postural trials under three different conditions: (1) eyes opened (EO): Participants stood with their eyes open; (2) eyes closed (EC): Participants stood with their eyes closed; (3) tandem condition (TC): Participants stood in a tandem stance, where one foot was placed directly in front of the other. Each trial lasted for 30 s and was followed by a 30 s rest period. Displacements of the CoP were recorded. Two postural control parameters were extracted from the CoP data. Firstly, mean sway area, which represents the area of the 95% confidence ellipse that encapsulates the sway of the CoP and is measured in square centimeters (cm^2^). Secondly, mean velocity of CoP displacements, which indicates the average velocity at which the CoP moved during the trial and is measured in millimeters per second (mm/s). These measurements provide insights into how participants’ postural control varies under different sensory conditions (EO, EC, and TC). The addition of TC allows for an evaluation of postural stability while standing in a more challenging stance.

#### 2.4.5. Electromyography Evaluation

Electromyography (EMG) data from the ankle joint muscles were recorded during MVC of PF and dorsal DF and postural control assessments. The data collection process utilized the Trigno^®^ Wireless Biofeedback System (Delsys Inc., Natick, MA, USA), with the EMG recording synchronized with the platform data during postural control trials. The sensors, comprising two pairs of silver bar contacts with a 10 mm interelectrode spacing, were positioned on the gastrocnemius medialis (GM), soleus (SOL), and tibialis anterior (TA) muscles of the dominant leg, following SENIAM recommendations. The raw EMG signals were then post-processed using Matlab software (Matlab R2013a, MathWorks, Natick, MA, USA). Data were collected over a 10 s period, starting from the 10th second of each trial (postural control test). The data underwent band-pass filtering in the range of 15–500 Hz using a second-order Butterworth digital filter to eliminate noise and movement interference. Subsequently, the data were rectified and smoothed using root mean square analysis (RMS) with a 20 ms window. For the MVC tests, a moving window with a width of 20 ms was employed to identify the peak RMS EMG activity resulting from the three MVC efforts for each type of contraction. RMS EMG data collected during postural control tests were then normalized to peak RMS EMG [2]. The normalized RMS of the GM (RMS GM), SOL (RMS SOL), and TA (EMG TA) from each postural control test were employed in this study.

### 2.5. Statistical Analysis

#### 2.5.1. Sample Size

The determination of the sample size was conducted using G*Power, a freely available software tool (version 3.1.9.4). The calculation was grounded in pre-defined parameters to control for both Type I error (alpha = 0.05) and Type II error (beta = 0.60), assuming a moderate anticipated effect size (r = 0.35). We used the variable as a reference for our sample calculation, following the approach of Ferhi et al. [30]. Under these conditions, it was determined that a minimum of 40 participants was necessary for the sample size.

#### 2.5.2. Statistical Procedures

Statistical analyses were performed using Statistica Software 13.0 (Software, Tulsa, OK, USA). The initial step involved an assessment of the normality of data distribution through Kolmogorov–Smirnov tests. For data that demonstrated a normal distribution, paired t-tests were used to compare results within the same group before and after the implementation of the TMP program. Furthermore, independent samples t-tests were employed to make comparisons between the IG and CG before and after the TMP program. In addition, we investigated the relationships between changes in postural parameters, neuromuscular parameters of the DF and PF, and muscle activity of the GM, SOL, and TA using Pearson’s correlation analysis. Data were presented as means and standard deviations, with the significance threshold set at *p* < 0.05.

## 3. Results

### 3.1. Participants

**Figure 1:**
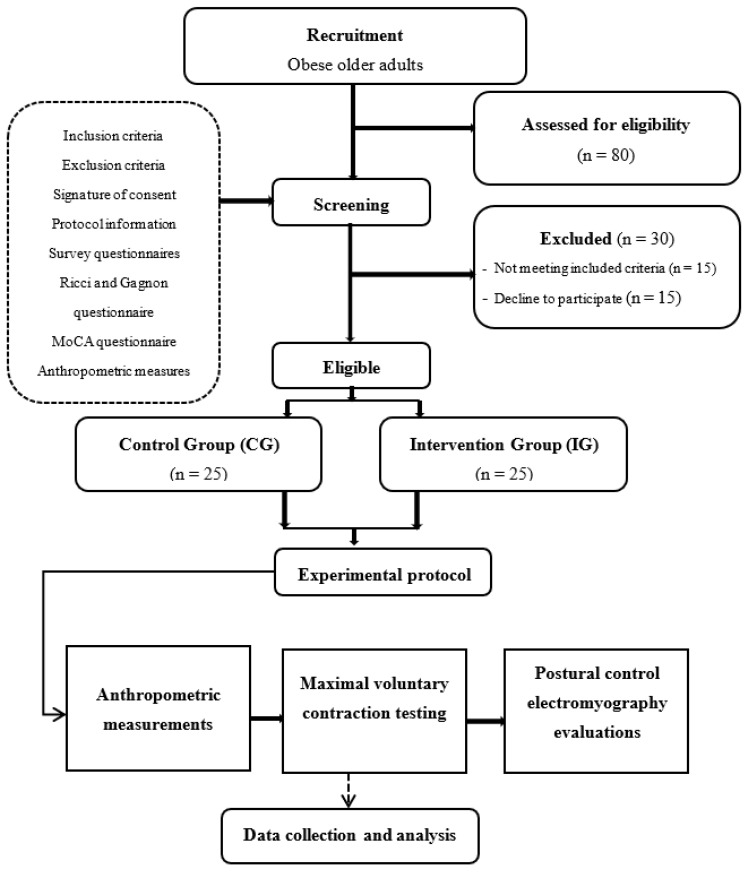
Flow diagram illustrating the experimental design.

Eighty volunteers were initially recruited but only sixty-five subjects met the eligibility criteria we had set. Fifteen individuals did not complete the study due to non-compliance with the study protocol (Figure 1). Ultimately, a cohort of fifty participants who successfully completed the entire study were randomly divided into two groups (Table 2): the CG (*n* = 25, mean age = 75.9 ± 5.4 years, mean BMI = 32.9 ± 2.3 kg/m^2^) and the IG (*n* = 25, mean age = 76.1 ± 3.5 years, mean BMI = 34.4 ± 4.0 kg/m^2^).

**Figure 2:**
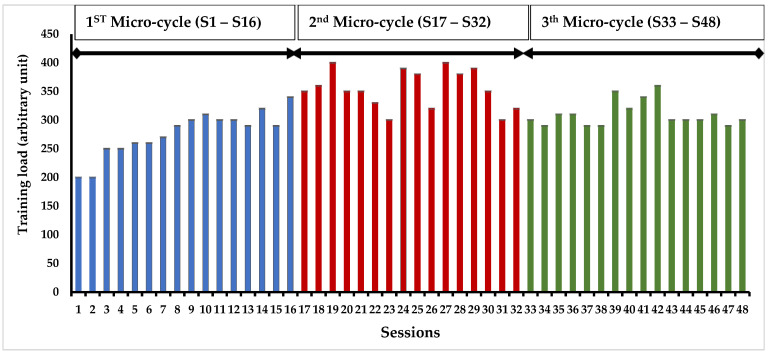
Progression of the training load in the intervention group over a four-month timeframe.

The progression of training load during the TMP program was monitored for participants (Figure 2). Analysis of the Ricci and Gagnon questionnaire indicated that both the control group (11.2 ± 2.5) and intervention group (10.7 ± 3.4) were inactive prior to commencing the TMP program.

### 3.2. Anthropometric Parameters

Table 2 presents results of the ROM, TUG and anthropometric parameters at baseline and after TMP intervention. At baseline assessment, there were no significant differences in anthropometric, ROM and TUG between the CG and IG.

However, after completing the TMP program, IG demonstrated significant decreases in time of standing up (ROM, −34.6%, *p* < 0.05) and time of TUG (−15.6%, *p* < 0.05). Moreover, the IG demonstrated a significant increase in LBM (+8.5%, *p* < 0.05) and a decrease in FBM (−17.7%, *p* < 0.05).

### 3.3. Maximal Voluntary Contraction Testing

**Figure 3:**
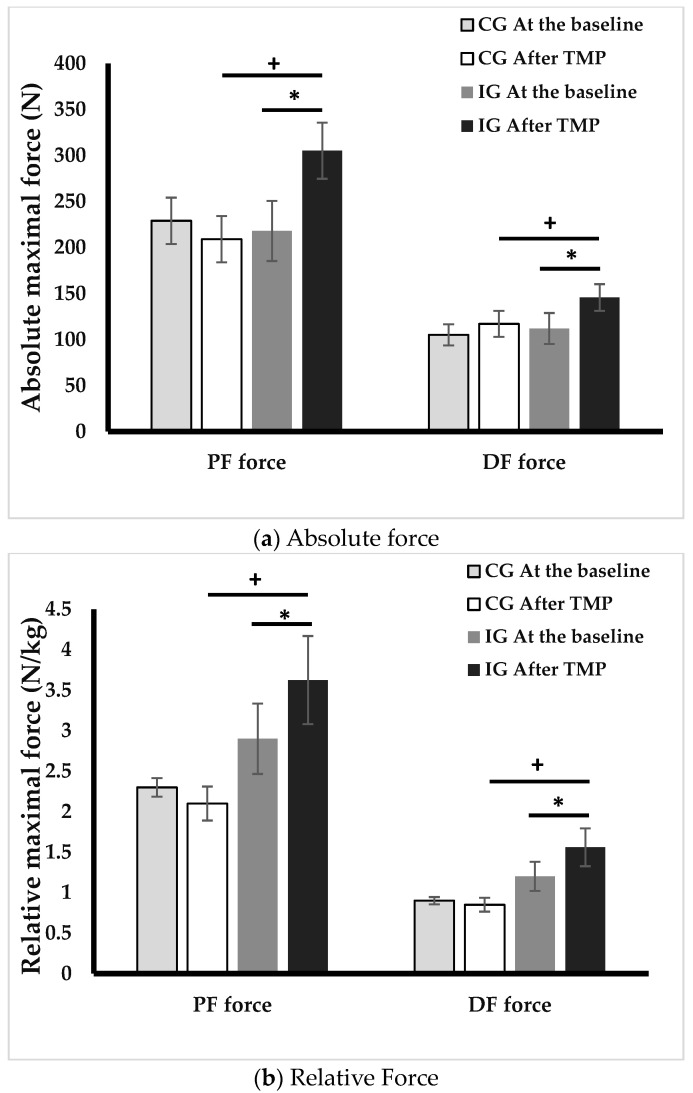
Absolute (**a**) and relative (**b**) forces of plantar and dorsal flexors at baseline and after intervention. PF: plantar flexors, DF: dorsal flexors, CG: control group, IG, intervention group, *: *p* < 0.05, difference between before and after TMP program, +: *p* < 0.05, difference between CG and IG.

The TMP intervention contributed to notable gains in both absolute and relative force of plantar (+40%, +25%, *p* < 0.05) and dorsal flexors (+51%, +30%, *p* < 0.05). Absolute and relative forces of plantar and dorsal ankle flexors were higher in IG compared with CG after the TMP program (*p* < 0.05). However, there were no significant differences between CG and IG at baseline (Figure 3).

### 3.4. Postural Control Evaluation

Table 3 represents CoP parameters during postural control tests at baseline and after TMP program. After the TMP intervention, IG showed a reduction in the CoP area in EO condition (−50%; *p* < 0.001), EC condition (−27%; *p* < 0.01), and TC (−34%; *p* < 0.01). A decrease in oscillation velocity was also observed in the EO (−27%; *p* < 0.01), EC (−19%, *p* < 0.01) and TC (−37%; *p* < 0.01) conditions following the TMP program. After the TMP program, CoP parameters in various conditions were higher in IG compared with CG (*p* < 0.05). Nonetheless, there were no significant differences between CG and IG at baseline (Table 3).

### 3.5. Electromyography Evaluation

**Figure 4:**
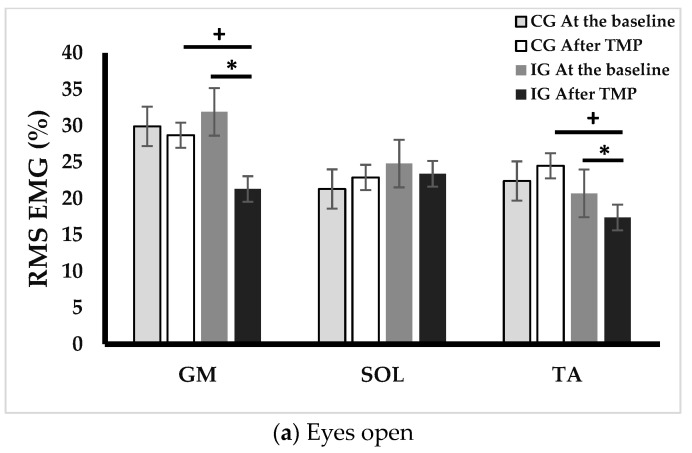

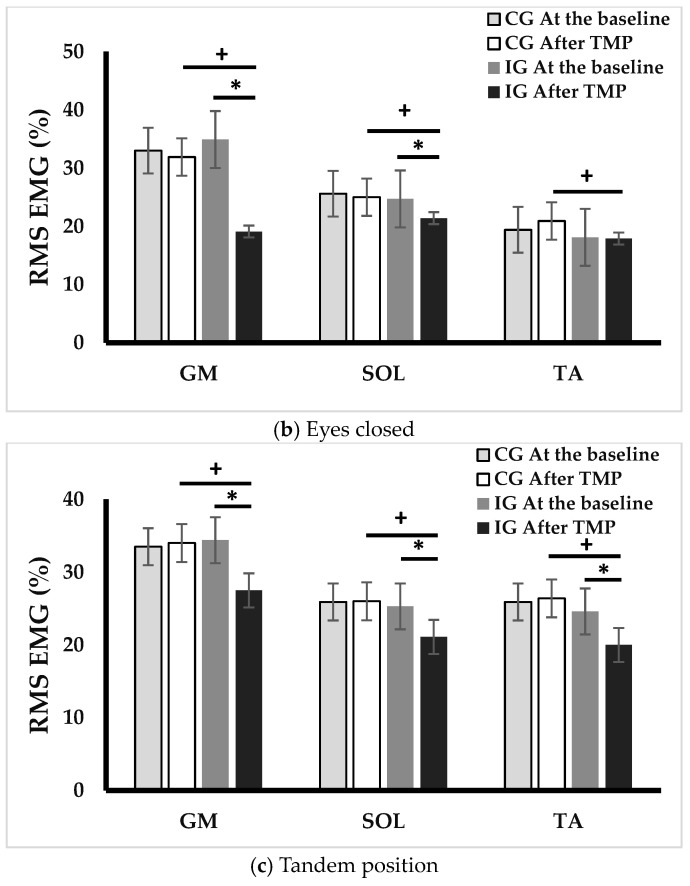
Changes in EMG activity across different postural control conditions. EO: eyes opened, EC: eyes closed, TC: tandem condition, GM: gastrocnemius medialis, SOL: soleus, and TA: tibialis anterior, CG: control group, IG, intervention group, *: *p* < 0.05, difference between before and after TMP program, +: *p* < 0.05, difference between CG and IG.

Figure 3 and Figure 4 represents ankle muscle activities during postural control tests at baseline and after TMP program. Following the TMP intervention, the IG showed significant reductions in GM activity in the EO (33.2%, *p* < 0.001), EC (29.9%, *p* < 0.01), and TC (20%, *p* < 0.01) conditions. SOL activity showed a decrease of 16.6% in both EC and TC conditions (*p* < 0.05). TA activity also manifested a decline in EO condition (15.9%, *p* < 0.05) and TC (17.4%, *p* < 0.05) conditions.

### 3.6. Pearson’s Correlation Analysis

Table 4 presents the correlation coefficients between postural control parameters and relative strength of ankle muscles. Ϫ PF was correlated with Ϫ CoP area and velocity in EO (r = 0.71, r = 0.69, *p* < 0.05, respectively) and EC (r = 0.62, r = 0.55, *p* < 0.05, respectively) conditions. Ϫ DF was correlated with Ϫ CoP area and velocity in EO (r = 0.42, r = 0.54, *p* < 0.05, respectively) and EC (r = 0.51, r = 0.49, *p* < 0.05, respectively) conditions.

## 4. Discussion

To our knowledge, this is the first study that has undertaken a comprehensive assessment of the effects of a 4 month physical activity program on the steady-state and proactive postural control, as well as on neuromuscular functions in older adults with SO. The findings from this pioneering research demonstrate that the TMP program resulted in significant enhancements in both static and proactive postural control. These improvements appear to be closely linked to a decrease in EMG activities and a notable increase in the force production capabilities of the muscles responsible for ankle joint mobility.

The results of this study demonstrate significant improvements in both steady-state (+34.6% in standing time) and proactive balance (−15.6% in time to achieve TUG) following participation in the TMP program. These improvements are further supported by notable reductions in various CoP displacement parameters, particularly a decrease in the area and the CoP velocity in different conditions. These findings collectively indicate an overall enhancement in postural control. These observations align with previous research that has highlighted the trainability of postural control functions in both normal-weight older adults [31,32], and obese older adults [8,28].

It has been previously suggested that the cumulative impact of age and obesity on postural control could be associated with synergistic effects arising from both sarcopenia and obesity [2,20]. In obese adults, postural control alterations can be attributed to several factors. Firstly, the constant load bearing associated with excess weight often leads to reduced plantar sensitivity due to the hyper-activation of plantar mechanoreceptors [33]. Secondly, managing the mechanical demands of increased body mass, particularly when distributed away from the rotational axis (as in the ankle joint, resembling an inverted pendulum model), results in an increased gravitational torque [18]. To counteract this torque, which acts along the anteroposterior axis, obese individuals must generate higher muscular torque to maintain an upright posture [34]. These challenges are further compounded by the presence of sarcopenia, a condition characterized by progressive alterations in neuromusculoskeletal, proprioceptive, and visual systems, collectively impairing postural control [35]. Given these considerations, we propose two hypotheses to explain the observed improvement in postural control among obese older adults with SO observed in our study. The first hypothesis relates to an increase in musculoskeletal capacities, possibly induced by strength exercises included in the TMP program. The second hypothesis relates to the enhancement of proprioceptive capacities resulting from posture and balance exercises. This hypothesis gains support from the improved postural parameters observed during tasks that require greater utilization of proprioceptive resources, particularly in the tandem position. These findings suggest that the TMP intervention, especially the balance exercises, effectively enhanced the proprioceptive capabilities of the participants. Furthermore, the improvements in postural parameters during this position were not correlated with changes in relative strength, indicating that the enhancement of postural control in older adults with SO likely relies on improved proprioceptive capacities rather than solely on increased strength.

Regarding the first hypothesis, the IG demonstrated a significant increase in relative ankle muscle force, with a 25% improvement in the PF and a 30% enhancement in the DF. The improvement in relative force was positively correlated with a decrease in the CoP parameters, specifically the area (r = 0.71) and velocity (r = 0.69) in the eyes open condition. This indicates that the improved strength of the plantar flexor muscles, achieved through muscle-strengthening exercises, may contribute to the improvement of balance in older adults with SO. Older adults with SO often struggle to generate sufficient force relative to their body mass during postural control, and this improvement in force production could be attributed to two possibilities: an increase in muscle mass and an improvement in neural mechanisms. In this study, an increase in LBM was observed without a change in BMI, explaining the modification of body composition following the TMP program. The absence of notable alterations in body composition can be ascribed to the substantial growth in LBM, displacing fat body mass [36]. This highlights the significance of regarding body composition as a vital gauge of exercise program effectiveness, particularly in older adults with significant frailty.

Regarding neuromuscular implications, one of the notable strengths of our study lies in its pioneering focus on the modifications in neuromuscular strategies of ankle muscles during postural control following the TMP program in individuals with SO. To our knowledge, this study is the first to delve into these specific neuromuscular adaptations within this population. In our investigation, we observed a significant reduction in ankle muscle activity during postural control trials in the IG group. GM activity decreased (from −20% to −33.2%), SOL activity (from −16.6% to −18.9%) and TA (from −15.9% to −17.4%). Importantly, postural control alterations in older adults with SO were previously attributed to increased ankle muscle activities, which were identified as a neuromuscular strategy to compensate for neuromuscular weakness and proprioceptive system degeneration [2,37]. However, increased ankle muscle activation leads to higher energy expenditure, increased costs, and early fatigue, ultimately elevating the risk of falls [25]. It is plausible to suggest that the TMP program improved the strategy of controlling ankle muscle activation during postural control, potentially reducing energy costs and, likely, early fatigue.

The impact of obesity on neuromuscular capacities can be elucidated by examining multiple factors, such as motor unit recruitment [38], intrinsic muscle properties [39,40], and systemic inflammation [41]. Moreover, adipose tissue in obese individuals’ functions as a dynamic endocrine organ, secreting an array of hormones and pro-inflammatory cytokines, like TNF-α, IL-1α, IL-6, and CRP [39]. On the other hand, obesity can exacerbate the loss of muscle mass—often referred to as sarcopenia—which is already compromised in older adults with SO [6]. The outcomes noted in this research, which encompass an augmentation in muscle mass and a decrease in neuromuscular activation, indicate that the TMP program, with its exercise regimens, appears to have a potential restorative impact on the mechanisms associated with neuromuscular system alterations resulting from the combined effects of obesity and sarcopenia. However, it is challenging to determine whether the reduction in ankle muscle activity is directly related to the improvement of the neuromuscular system or to a decreased demand associated with postural regulation in older adults with SO. This necessitates further investigations to better understand the underlying mechanisms of neuromuscular system modification, postural control regulation, and the effects of physical activity on the reversibility and trainability of the neuromuscular system.

### Limitations and Perspectives

This study acknowledges several limitations that warrant consideration. Firstly, results observed in this study may restrict the generalizability of our findings, particularly given the heterogeneity often observed in older adults and obese individuals. These populations can sometimes present a complex interplay of factors, resulting in characteristics that may not be wholly reflective of those in our study. Furthermore, our study predominantly centered on the musculature surrounding the ankle joint, disregarding potential impacts of surplus adipose tissue in the knee and hip joint areas on EMG signals. For a more thorough evaluation of neuromuscular aspects, the inclusion of 3D gait analysis to assess joint moments and ranges of motion across three joints (hip, knee, and ankle) would be advantageous.

## 5. Conclusions

This study unveiled significant enhancements in both steady-state and proactive postural control following a comprehensive physical activity program. These improvements were coupled with remarkable reductions in various parameters associated with the CoP displacement, indicating an overall advancement in postural control regulation. Importantly, these improvements were not solely linked to increased muscular strength but also to the development of enhanced proprioceptive capacities, potentially fostered through balance exercises within the program. These findings highlight the reversibility of neuromuscular system alterations associated with the synergistic effects of sarcopenia and obesity, emphasizing the trainability of postural control regulation within this population. By incorporating these insights into clinical practice and public health strategies, we can strive to optimize the health and well-being of older adults dealing SO.

## 6. Recommendations

Several critical factors should be considered when implementing concurrent exercise training for vulnerable populations. First, the quantitative aspects of the program should be rooted in individuals’ self-perceived exertion levels. This approach enables the establishment of the most suitable exercise intensity for each participant. By avoiding excessive fatigue, negative consequences can be minimized, and an appropriate intensity level is can be maintained. Furthermore, regular assessments conducted throughout the program are essential. These assessments are instrumental in monitoring progress and facilitating adjustments to the training load or exercise regimen as required. Beyond the physical aspects, it is equally crucial to account for psychological and social factors, such as motivation. Creating a nurturing and engaging environment within the program is paramount for cultivating sustained motivation among older adults. This support system encourages adherence to the program, thereby promoting the long-term advantages of physical activity for individuals with significant frailty.

## Figures and Tables

**Table 1:** Description of the TMP intervention.

Intervention Description and Replication (TIDieR) Guidelines	
Name	The TMP program.
Why	To enhance steady-state and proactive postural control in older adults with SO (*n* = 25).
Materials	Diverse range of physical materials: chairs, balls, markers, slats, cups, hoops, elastic bands, and weighted bags, foam rollers, balance boards, resistance tubes and bands, exercise mats, medicine balls, step platforms, cones, kettlebells, and step platforms.
Procedures	The TMP program was conducted over a 16-week duration, comprising three 60-min sessions per week, for a total of 48 sessions over the course of the intervention. Each session adhered to a structured protocol, commencing with a 10-min warm-up. The central component of each session encompassed motor skill exercises and exercises emphasizing strength and posture, with the duration determined by the prescribed training volume.Motor skill exercises: Zigzag cone walking: A series of cones is set up in a zigzag pattern and participants encouraged to walk through them, changing direction with each turn. Obstacle course walk: An obstacle course is created with hurdles, cones, and other barriers. Participants must navigate through the obstacles, promoting agility and precise footwork. Ladder agility drills: An agility ladder is laid on the ground and participants encouraged to walk through it, changing direction with each step. Direction change walking: Participants walk in a straight line and, on a signal, change direction at a 90 degree angle, alternating between left and right turns. Obstacle relay race: A relay race is organized in which participants must navigate an obstacle course with zigzag sections, hurdles, and direction changes. Zigzag Ball Passing: Participants walk a zigzag path while passing a ball to a partner. The path can include cones or markers to change direction. Balance board zigzag walk: A balance board or balance beam with zigzag sections is used. Participants must walk along the zigzag path while maintaining balance, improving stability and coordination. Cone weaving: Cones are set up in a zigzag pattern and participants encouraged to weave in and out of them while walking. Strengthening exercises: Calf Raises: Participants stand behind a sturdy chair or use a wall for support, they then rise up onto their toes and lower their heels down. Toe Taps: Participants sit in a chair with their feet flat on the floor. They then lift their toes and tap them down, keeping their heels on the ground. Seated Leg Extensions: Participants sit in a sturdy chair, before extending one leg straight in front, holding for a few seconds, and then lowering it. Mini Squats: Participants stand in front of a chair and perform shallow squats, gently lowering their hips toward the chair without sitting down completely. Seated Leg Lifts: Participants sit in a chair and lift one leg straight out in front, holding for a few seconds and then lowering it back down. Bridge Exercise: Participants lie on their back with their knees bent and feet flat on the floor. They then lift their hips off the ground by pushing through their heels. Leg Raises: While holding onto a chair or countertop for support, participants lift one leg behind themselves while keeping it straight. They then lower it back down. Hip Abduction with Resistance Band: Participants place a resistance band around their ankles while sitting in a chair. They then push their legs outward against the resistance of the band. Posture exercises: Single-Leg Balance with Eyes Closed: Participants stand on one leg and, for an added challenge, close their eyes. Heel-to-Toe Walking with Eyes Closed: Similar to heel-to-toe walking, but with eyes closed. Balance Board or Wobble Board: Standing on a balance or wobble board challenges proprioception. Tandem Stance: Participants stand with one foot directly in front of the other, heel to toe. Toe Tapping: While seated, participants must tap their toes on the floor in different patterns, such as side-to-side or in circles. Ball Toss: Participants toss a soft ball between hands while standing on one leg. Reaching and Bending: While standing on one leg, participants must reach for objects on the ground or across their body. Sensory Walk: Participants walk on different surfaces, such as grass, sand, or gravel, to stimulate proprioceptors in the feet. Pick Up Objects with Toes: Participants must practice picking up small objects from the floor using their toes. This exercise enhances foot and ankle proprioception.
Who	Conducted by a specialized kinesiologist in adapted physical activity.
How	Collective sessions.
Where	In the rehabilitation spaces.
How much	Forty-eight sessions. Each session had a duration of 60 min. The design of the exercise types within the program was customized to suit the training load of each session and was based on predefined training intensity and volume for individual sessions. Each exercise regimen included 1 to 5 sets, with repetitions varying from a minimum of 3 to a maximum of 15 per set.
Tailoring	Modifications to the training intensity were assessed after each session using the Rating of Perceived Exertion (RPE) scale, as outlined in the study by Ferhi et al. [30].
Modifications	Adjustments were made during each session, following the guidelines provided in the study by Ferhi et al. [30].
How planned	The TMP program comprised three micro-cycles, as outlined in Ferhi et al. [30].
Actual	All participants completed the TMP program.

**Table 2:** Differential analysis of balance and anthropometric characteristics between the control and intervention groups at baseline and after the TMP program.

	CG *n* = 25	IG *n* = 25		CG *n* = 25	IG *n* = 25	
	At Baseline		*p*-Value	After the Intervention		*p*-Value
Anthropometric characteristics	Anthropometric parameters					
Age (years)	75.9 ± 5.4	76.3 ± 3.5	NS	76.3 ± 5.4	76.7 ± 3.5	NS
Body height (cm)	163.2 ± 4.2	165.7 ± 4.9	NS	163.2 ± 4.2	165.7 ± 4.9	NS
Body mass (kg)	92.1 ± 6.4	94.2 ± 5.1	NS	90.9 ± 5.4	92.6 ± 6.4 *+	NS
BMI (kg/m^2^)	34.7 ± 2.3	34.5 ± 4.0	NS	33.4 ± 2.4	31.9 ± 1.4	NS
Body fat (%)	40.0 ± 4.3	39.0 ± 4.5	NS	40.4 ± 7.1	32.6 ± 4.5 *+	<0.01
FBM (kg)	31.9 ± 3.6	36.7 ± 5.5	NS	36.8 ± 3.6	30.2 ± 5.5 *+	<0.05
LBM (kg)	60.1 ± 4.2	57.5 ± 6.4	NS	54.2 ± 4.2	62.4 ± 3.4 *+	<0.05
Waist circumference (cm)	94.8 ± 4.9	89.3 ± 4.8	NS	94.2 ± 4.1	85.4 ± 4.9	NS
Hip circumference (cm)	92.0 ± 6.5	98.9 ± 4.6	NS	92.4 ± 6.1	96.5 ± 6.0	NS
Hand grip (N)	13.7 ± 3.2	13.0 ± 2.5	NS	13.3 ± 3.0	16.3 ± 3.3 *+	<0.05
Maximal gait speed (m/s)	0.8 ± 0.2	0.7 ± 0.3	NS	0.8 ± 0.2	1.1 ± 0.4 *+	<0.05
Tests	Balance parameters					
Time Up and Go (s)	12.9 ± 1.9	14.1 ± 2.1	NS	13.1 ± 1.64	11.9 ± 1.34	<0.05
Romberg test (s)	13.7 ± 3.2	13.3 ± 3.0	NS	14.3 ± 2.37	17.9 ± 2.32	<0.01

CG: control group; IG: intervention group; BMI: body mass index; FBM: fat body mass; LBM: lean body mass; ASM: skeletal muscle mass index; ASM: appendicular skeletal muscle mass; *: difference between groups; +: difference between baseline and after intervention in IG, NS: no significant difference.

**Table 3:** Changes in center of pressure parameters before and after 4 month TMP intervention across different conditions.

			Intervention Group			
Conditions	Group	CoP Parameters	Baseline	After TMP	Δ (%)	*p*-Value
EO	IG	Area (cm^2^)	8.4 ± 3.2	5.2 ± 2.9 *+	−26	<0.001
	CG		8.1 ± 5.4	8.6 ± 7.2	+6	NS
	IG	Velocity (mm/s)	24.9 ± 10.6	17.0 ± 4.2 *+	−31	<0.01
	CG		22.9 ± 8.6	23.4 ± 6.2	+3	NS
EC	IG	Area (cm^2^)	10.7 ± 3.7	7.8 ± 3.6 *+	−27	<0.01
	CG		10.1 ± 4.9	10.5 ± 6.6	+4	NS
	IG	Velocity (mm/s)	32.6 ± 10.2	26.5 ± 7.6 *+	−19	<0.05
	CG		29.1 ± 8.2	31.6 ± 8.9	+9	NS
TC	IG	Area (cm^2^)	24.8 ± 5.1	16.4 ± 4.5 *+	−34	<0.01
	CG		27.1 ± 9.1	25.9 ± 11.1	−4	NS
	IG	Velocity (mm /s)	54.9 ± 14.1	34.4 ± 6.3 *+	−37	<0.05
	CG		53.4 ± 10.1	55.9 ± 12.1	+5	NS

EO: eyes opened, EC: eyes closed, TC: tandem condition, CG: control group, IG, intervention group, *: *p* < 0.05, difference between before and after TMP program, +: *p* < 0.05, difference between CG and IG.

**Table 4:** Pearson’s correlation analysis.

	Intervention Group					
	EO		EC		TC	
	Ϫ Area	Ϫ Velocity	Ϫ Area	Ϫ Velocity	Ϫ Area	Ϫ Velocity
Ϫ PF relative	0.71 *	0.69 *	0.62 *	0.55 *	0.37	0.39
Ϫ DF relative	0.42 *	0.54 *	0.51 *	0.49 *	0.39	0.42 *

FP: plantar flexors, DF: dorsiflexors, EO: eyes open, EC: eyes closed, TC: tandem condition, *: *p* < 0.05.

## Data Availability

The data supporting the reported results of this study will be published on the Pan African Clinical Trials Registry after the publication of the article. Interested parties can access the data by referring to the registry once it becomes available.
